# A Novel Fusion Protein System for the Production of Nanobodies and the SARS-CoV-2 Spike RBD in a Bacterial System

**DOI:** 10.3390/bioengineering10030389

**Published:** 2023-03-22

**Authors:** Dóra Nagy-Fazekas, Pál Stráner, Péter Ecsédi, Nóra Taricska, Adina Borbély, László Nyitray, András Perczel

**Affiliations:** 1 Laboratory of Structural Chemistry and Biology, Institute of Chemistry, Eötvös Loránd University, Pázmány Péter sétány 1/A, H-1117 Budapest, Hungary; 2 Hevesy György PhD School of Chemistry, Institute of Chemistry, Eötvös Loránd University, Pázmány Péter sétány 1/A, H-1117 Budapest, Hungary; 3 ELKH-ELTE Protein Modeling Research Group, Eötvös Loránd Research Network (ELKH), Institute of Chemistry, Eötvös Loránd University, Pázmány Péter sétány 1/A, H-1117 Budapest, Hungary; 4 Department of Biochemistry, Eötvös Loránd University, Pázmány Péter sétány 1/C, H-1117 Budapest, Hungary; 5 MTA-ELTE Lendület Ion Mobility Mass Spectrometry Research Group, Department of Analytical Chemistry, ELTE Eötvös Loránd University, Pázmány Péter sétány 1/A, H-1117 Budapest, Hungary

**Keywords:** camelid immunoglobulins, nanobody, bacterial expression, well-folded SARS-CoV-2 S protein RBD variants

## Abstract

Antibodies are key proteins of the immune system, and they are widely used for both research and theragnostic applications. Among them, camelid immunoglobulins (IgG) differ from the canonical human IgG molecules, as their light chains are completely missing; thus, they have only variable domains on their heavy chains (VHHs). A single VHH domain, often called a nanobody, has favorable structural, biophysical, and functional features compared to canonical antibodies. Therefore, robust and efficient production protocols relying on recombinant technologies are in high demand. Here, by utilizing ecotin, an *Escherichia coli* protein, as a fusion partner, we present a bacterial expression system that allows an easy, fast, and cost-effective way to prepare nanobodies. Ecotin was used here as a periplasmic translocator and a passive refolding chaperone, which allowed us to reach high-yield production of nanobodies. We also present a new, easily applicable prokaryotic expression and purification method of the receptor-binding domain (RBD) of the SARS-CoV-2 S protein for interaction assays. We demonstrate using ECD spectroscopy that the bacterially produced RBD is well-folded. The bacterially produced nanobody was shown to bind strongly to the recombinant RBD, with a K_d_ of 10 nM. The simple methods presented here could facilitate rapid interaction measurements in the event of the appearance of additional SARS-CoV-2 variants.

## 1. Introduction

Antibodies (Ab), and among them immunoglobulin G (IgG), are key proteins of our immune system. Their target specificity makes them attractive for diagnostic and therapeutic applications. Therefore, the development of their recombinant forms (rAbs), especially the different types of antibody fragments (e.g., scFab and ScFv), which have simpler structure and higher stability, has been at the center of biotechnology research in recent decades [1,2,3]. In this respect nanobodies of camelid origin are of especially high interest due to their high antigen binding and neutralizing capability combined with small size and high stability [4].

Camelid antibodies are very similar to human IgG-type antibodies, but their structure is devoid of light chains [5], meaning they have only heavy chain variable domains (VHHs) [4]. A single copy of this variable domain is often called a nanobody. Because of the beneficial biophysical properties and the low cost of production, nanobodies can be easily and quickly used against different diseases [6]. As an example, trivalent nanobodies such as the mNb6-tri retain function after heat treatment, lyophilization, and aerosolization that enables aerosol-mediated delivery to the airway epithelia [7].

Nanobodies can be effective tools against newly emerging diseases, such as the coronavirus disease COVID-19, which first appeared in Wuhan, China in December 2019 and subsequently spread worldwide. By copying the variable regions of natural antibodies, different nanobody constructs with ultra-high neutralization potency can be easily engineered and produced in large quantities and then used against different SARS-CoV-2 variants [8]. As an example, the sequences of the bivalent V_H_-Fc ab8, which binds with high avidity to the membrane-associated S glycoprotein of SARS-CoV-2 and directly interferes with its binding to the human angiotensin-converting enzyme 2 (ACE2), can be utilized for this purpose [9]. Another example is the H11-H4 nanobody, which has been shown by molecular dynamics simulations to have high neutralizing ability against the alpha, kappa, and delta variants of SARS-CoV-2 [10]. The application of nanobody-based drug development against new diseases such as SARS-CoV-2 is on the rise, and the recently demonstrated high efficiency of the PiN-21 nanobody-based intranasal drug to prevent and treat SARS-CoV-2 infection may provide a convenient and cost-effective option to mitigate the ongoing pandemic [11].

The more favorable properties of nanobodies also allow more efficient production in other hosts besides mammalian cells such as yeast, fungi, insect cells, or even bacteria [4,12,13]. However, each of these expression systems has its own limitations. Production in yeast could result in reduced or lost nanobody functionality because of the presence of yeast-specific mannose sugars [12]. The expression of nanobodies by fungi has other drawbacks too, namely the presence of fungi-secreted proteases that could partially degrade the products. Since nanobodies contain conserved disulfide bridges that require an oxidative environment for proper folding, the expression of nanobodies in the cytoplasm of conventional bacterial cells such as *BL21* results in inclusion bodies that require complex purification and refolding protocols [14]. The best way to express mammalian proteins is to use mammalian cells; however, this is the most expensive, low-yielding, and time-consuming method. The most similar system is expression using insect cells; however, this has drawbacks as well, such as the time-consuming cloning procedure, the expensive media, and the fact that glycosylation is different from that of mammalian systems. This can result in improper maintenance of epitopes on protein [13].

Because of this, using specific strains with a more oxidative cytoplasm (*Shuffle* or *Rosetta-gami*) or expressing nanobodies in the periplasm of *E. coli* is a more favorable method for this purpose [12,15]. The main advantage of the latter is the ease of the required purification protocols, while the most serious disadvantage would be the unpredictable yields that could depend on the type of signal sequence used or on the composition of the nanobodies. The conventional periplasmic expression protocol of nanobodies is to target the *C*-terminal His-tagged single domain of the antibody into the periplasm using an *N*-terminal signal sequence. Note here that the expression of the nanobodies using conventional fusion partners (MBP or TrxA) was also described [16,17], but in these cases, due to the large molecular weight of the fusion partner, the yield of the nanobodies after enzymatic cleaving was unfavorably low. These uncertainties and difficulties show that the expression protocols for periplasmic production can and should be improved to be more robust and cost effective and to support the wider use of nanobodies.

In the present work, SARS-CoV-2-neutralizing nanobodies (Nb20, ab8, H11-H4, and VHH72—sequences are in the Supplementary Information) [9,18,19,20] were used as models for the development of a novel system that allows an easy, fast, reliable, and cost-effective way of expressing nanobodies in the *E. coli* periplasm or in the cytoplasm using ecotin as a fusion partner. We also set out to produce the receptor-binding domain (RBD) of the SARS-CoV-2 spike protein to carry out interaction studies with the neutralizing antibodies.

## 2. Materials and Methods

### 2.1. Construction of Expression Plasmids

The ecotin gene with its *N*-terminal signal sequence followed *C*-terminally with a His-tag, a thrombin cleavage site, and a pET-32b-derived multiple cloning site was synthesized as a double-stranded gene fragment (Integrated DNA Technologies (IDT)). This gene fragment was cloned using restriction ligation into two different vectors (pMAL (Tac promoter, NdeI-XhoI) and pET-32b (T7 promoter, NdeI-XhoI)) to test the effect of the different promoters.

The genes of each target nanobody were also synthesized as a double-stranded gene fragment (IDT) and ligated using the restriction enzyme pair (BamHI-XhoI) into the two vector types. An *N*-terminal His-tagged vector series with different promoters (Tac and T7) was also constructed to test nanobody expression directly in the cytoplasm (Supplementary Information).

The genetic sequence of the wild-type form of the spike RBD was codon optimized to an *E. coli* strain and was fused to the MBP gene and cloned into the pET32Mthr vector [21]. (Supplementary Information).

The genetic sequence of the wild-type form of the full-length spike protein was cloned into a modified pBlueScript vector (pL2, containing PiggyBac recognition sites) with CAG promoter and a thrombin-cleavable *C*-terminal His-tag (Supplementary Information).

DH5α competent cells (Subcloning Efficiency DH5α competent cells ThermoFisher (Waltham, MA, USA), cat. No. 18265017) were used for plasmid cloning procedures and transformed by heat shock transformation following the manufacturer’s instructions. Transformed cells were then selected on LB agar containing ampicillin (100 μg/L).

### 2.2. Nanobody Expression and Purification

The expressed nanobody plasmids were transformed into different *E. coli* strains (*C43(DE3), Shuffle-T7*, *HB2151,* and *BL21(DE3*)). *C43(DE3) E. coli* (Sigma-Aldrich (St. Louis, MO, USA), cat. No. CMC0019), *Shuffle-T7 E. coli* (New England Biolabs (Ipswich, MA, USA), cat. No. C3026J), *HB2151 E. coli* (Nova Lifetech (Singapore), cat. No. S0122), and *BL21(DE3) E. coli* (New England Biolabs, cat. No. C2527H) were used as hosts for protein expression and purification studies.

The cells were grown at 37 °C overnight on LB agar plates containing ampicillin (100 μg/L). A 50 mL preculture with LB medium containing colonies from the LB agar plate and ampicillin (100 μg/mL) was prepared and grown for 3 h at 37 °C at 180 rpm shaking. For protein production, 2YT cultures containing 2 g/L of glucose were inoculated with 1% from the preculture and grown at 37 °C. Induction of recombinant protein synthesis was initiated at an OD_600_ of 0.8 by adding 0.5 mM IPTG. Incubation temperature during protein expression overnight (~12 h) was 30 °C at 180 rpm shaking.

The periplasmic fraction of the cells was prepared by the osmotic shock method. Cells were harvested at 4500× *g* for 10 min at 4 °C. The pellet was resuspended in 50 mL/L of ice-cold hypertonic solution (30 mM Tris, 20 *w*/*v*% sucrose, 1 mM EDTA, pH = 8). After 30 min of incubation on ice, with gently shaking in every 5 min, the suspension was centrifuged for 20 min at 7600× *g* at 4 °C. The supernatant was collected and kept at 4 °C (first periplasmic fraction), and the cells were resuspended in the same volume of ice-cold hypotonic solution (5 mM MgCl_2_) and held on ice for 30 min with gentle shaking every 5 min. After 20 min centrifugation at 7600× *g* and 4 °C, the supernatant was collected (second periplasmic fraction). The pellet was resuspended in buffer A (300 mM NaCl, 50 mM Na_2_HPO_4_, pH = 8) and sonicated (5 times for 2 min, 50% grade) on ice (cytoplasmic fraction). After centrifugation (23,000× *g*, 20 min, 4 °C) the supernatant was collected as the cytoplasmic fraction. The periplasmic fractions were dialyzed in 4 L of buffer A overnight at 4 °C.

The cytoplasmic and the dialyzed periplasmic fractions were applied to a 5 mL HisTrap FF column (cytiva, Uppsala, Sweden) equilibrated with 5 column volumes (CV) of buffer A. The sample was applied at a flow rate of 0.8 mL/min, then the column was washed with 5 CV of buffer A. The elution was performed by an isocratic gradient with 5 CV of buffer B (buffer A + 250 mM imidazole, pH = 8) at a rate of 4 mL/min.

The collected fractions were pooled and dialyzed in 4 L of PBS buffer. After dialysis, the fusion protein was cleaved by thrombin protease (0.01 μL/μg) overnight. The purification of the target VHH was performed by a second HisTrap chromatography purification step, where the flow-through fractions were collected. The sample was then concentrated using a 10 kDa ultrafiltration device and purified by size-exclusion chromatography (Superdex 75 10/300 GL (GE Healthcare, Uppsala, Sweden)). The fractions from the different purification steps were analyzed by SDS-PAGE assessing the correct molecular weight, and after SEC, it was also confirmed by HPLC-MS (Supplementary Information, Figures S1–S3).

### 2.3. RBD Domain Expression and Purification

The MBP-RBD plasmid was transformed into the *E. coli Shuffle-T7* strain. Cells were grown at 37 °C on LB agar plates containing ampicillin (100 μg/L). Then, 50 mL preculture with LB medium containing colonies from the LB agar plate and ampicillin (100 μg/L) was prepared and grown for 3 h at 37 °C at 180 rpm shaking. For protein production, the 2YT cultures containing 2 g/L of glucose were inoculated with 1% of the preculture and grown at 37 °C. Induction of recombinant protein synthesis was initiated at OD_600_ of 0.8 by adding 0.5 mM IPTG. Incubation temperature during protein expression overnight (~12 h) was 30 °C at 180 rpm shaking. Cells were harvested (4500× *g* 20 min, 4 °C). The pellet was resuspended in 50 mL/L of resuspension buffer (50 mM Tris, 300 mM NaCl, pH = 8.0) and sonicated 5 times for 2 min 50% on ice. After sonication, the suspension was centrifuged (23,000× *g*, 20 min, 4 °C).

The cytoplasmic fraction was applied to an MBPTrap HP column (GE Healthcare, Uppsala, Sweden) equilibrated with 5× volume of CV resuspension buffer. The sample was applied at a flow rate of 0.8 mL/min, and the column was washed with 3 CV of the same buffer. The elution was performed by the isocratic mode with 5 CV of the elution buffer (resuspension buffer + 40 mM maltose, pH = 8.0) at a rate of 4 mL/min, and fractions containing the protein of interest with the fusion protein were collected.

The possibly incorrect disulfide bonds in the RBD domain were reduced by adding 10 mM DTT to the collected fractions and incubating the mixture for two days at 37 °C. To avoid non-specific protease cleaving, 1 mM EDTA was also added. Then the protein was refolded by the slow-drop method into the continuously stirred refolding mixture (50 mM Tris, 150 mM NaCl, 1 mM EDTA, 10 mM GSH, 1 mM GSSG, pH = 8) and was incubated at 20 °C for two days. Then the precipitations were removed by centrifugation (23,000× *g*, 20 min, 4 °C) and filtration (0.45 μm). The target protein was ultra-filtrated using a stirrer ultrafiltration device (Millipore (Burlington, MA, USA)).

For purification of the fusion protein, a HiLoad Superdex 16/600 75 pg column (GE Healthcare, Uppsala, Sweden) was equilibrated with 1.25 CV of resuspension buffer. Next, 3 mL of protein was injected, and 1.5 CV of isocratic elution was performed at a 0.5 mL/min flow rate. The elution was recorded at 280 and 220 nm. The fractions from the different purification steps were analyzed by SDS-PAGE to assess the correct molecular weight.

For control measurements, the full-length His-tagged S-protein and the His-tagged RBD domain were expressed in CHO Express cells (ECACC General Cell Collection (Salisbury, UK): CHO-K1, cat. No. 85051005) for 12 days at 37 °C. The cell medium was collected and purified by using Ni-IMAC by using a PBS buffer system.

### 2.4. LC-MS Analysis

Reverse-phase LC-MS analyses of intact proteins were performed on a Waters Acquity I-Class UPLC system coupled directly to a high-resolution hybrid quadrupole-time-of-flight mass spectrometer (Waters Select Series Cyclic IMS; Waters Corporation (Milford, MA, USA)). Samples were analyzed using a Waters Acquity BEH 300 C4 UPLC column (2.1 × 150 mm, 1.7 μm) under the following parameters: mobile phase “A”: 0.1% trifluoroacetic acid in water; mobile phase “B”: 0.1% trifluoroacetic acid in acetonitrile; flow rate: 300 μL/min; column temperature: 60 °C; gradient: 2 min: 5% B, 8 min: 55% B, and 8.5 min: 90% B. UV detection was performed at 220 nm and 280 nm. The spectrometer was operated in ESI positive V mode. Leucine encephalin was used as a lock mass standard. The *m*/*z* range was 900–2000. Deconvolution was performed by MaxEnt 1 software (MassLynx v4.2, Waters Corporation).

### 2.5. Biolayer Interferometry Measurements

The S-protein RBD and nanobody interaction was studied using an Octet K2 (Sartorius) system with Anti-Penta-HIS biosensor tips. Nanobodies were dialyzed in PBS to minimize signal errors caused by the buffer difference. In the first setup, the full-length S-protein (20 μM solution) was loaded to the surface, and binding of the different nanobodies to the S-protein in decreasing concentrations was measured. The association and dissociation curves were analyzed by using the Octet System Software (Data Analysis HT 10.0.3.7) and the program Origin.

### 2.6. Electronic Circular Dichroism Measurements

FUV-ECD spectra were recorded on a Jasco J810 spectrophotometer by using a 1.0 mm path length cuvette with protein concentrations of 16 μM. Data accumulation was performed over the range of 195–260 nm, with 0.2 nm step resolution at a scan rate of 50 nm/min with a 1 nm bandwidth. Spectral accumulations were resolved at 25 °C. The temperature was controlled by using a Peltier-type heating system. From each protein spectrum, a baseline spectrum was subtracted, which is defined as the spectrum of the pure solvent. Raw ellipticity units [Θ]_MR_ were processed similarly.

NUV-ECD spectra were recorded on the same spectrophotometer by using a 10 mm path length cuvette with protein concentration of 8–16 μM. Data accumulation was performed over the range of 250–350 nm, with 0.2 nm step resolution at a scan rate 50 nm/min with a 1 nm bandwidth. Spectral accumulations were resolved at 25 °C. The temperature was controlled by using a Peltier-type heating system. From each protein spectrum, a baseline spectrum was subtracted, which is defined as the spectrum of the pure solvent. Raw ellipticity units [Θ] were processed similarly.

## 3. Results and Discussion

### 3.1. Design of SARS-CoV-2 Neutralizing Nanobodies and SARS-CoV-2 Spike RBD Variants

The first aim of this project was to construct a suitable expression system for the production of single-domain antibodies with good yield and correct fold using ecotin as a fusion partner. Ecotin is a small (16 kDa) dimeric serine protease inhibitor of *E. coli* that is expressed constitutively during bacterial growth into the periplasm [22]. Taking advantage of this, it was previously reported that ecotin works well as a translocator in bacterial expression systems for recombinant protein expression [23,24]. We assumed that using ecotin would provide some advantages because it is a native, periplasmic *E. coli* protein; thus, (*i*) periplasmic translocation would not depend on the targeted nanobody’s sequence and folding properties, (*ii*) the yield would be reliable and high, and (*iii*) in an appropriate strain, the fusion protein could be expressed and folded in the cytoplasm with an expected increased yield.

**Figure 1:**
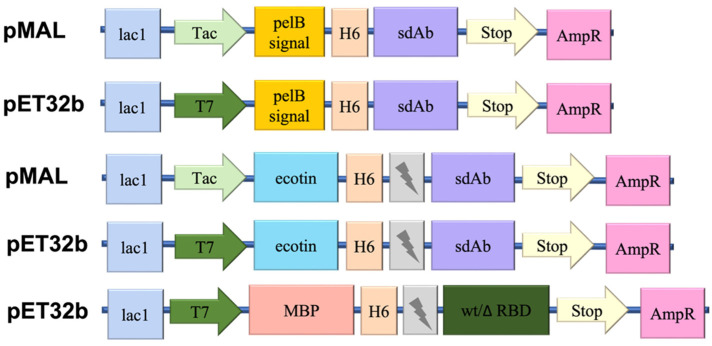
The construction scheme of the pMAL and pET32b vectors containing promoter regions with pelB, ompA, or without a signal sequence and with the ecotin fusion protein, histidine tag, thrombin cleavage site (lightning bolt symbol), and different single domain antibody (sdAb) sequences. The bottom scheme is another pET32b vector containing an MBP fusion protein instead of the ecotin version and containing the wild-type or delta variant of the SARS-CoV-2 spike protein RBD sequences.

To test these advantages of the ecotin fusion, two different bacterial vectors were constructed with different promoter regions: the most common T7 and the stronger Tac promoter. As a control, we tried to express the selected nanobodies with and without pelB and ompA signal sequences to test both the periplasmic and direct cytoplasmic expression, respectively. We tested these vector constructs (Figure 1) in different strains such as a conventional *BL21(DE3)* strain, the *C43(DE3)* strain for successful periplasmic expression, the *Shuffle-T7* strain that mimics the periplasmic environment in the cytosol, allowing disulfide bond formation in the protein, and the *HB2151* strain to reach higher yield in the cytoplasm [25,26,27].

An additional goal of the project was to create a suitable expression system to produce a biologically active antigen with good yield for testing the neutralizing nanobodies in a fast and accurate way. Although the most convenient method of recombinant protein production is rapid and economical bacterial production [28,29], efficiently expressing the RBD is challenging. The RBD consists of β-sheets and five disulfide bonds stabilizing its structure [30,31]. Moreover, the presence of the β-sheets also increases the aggregation tendency of the protein [32]. There are examples of bacterial expression of recombinant SARS-CoV-2 spike RBD, including a refolding method to achieve biologically active protein, although these have low and unpredictable yields [21]. To avoid the expected problematic periplasmic targeting, we used maltose-binding protein (MBP) as a fusion partner to ensure the correct 3D-protein fold and a stable and reliable yield of the recombinant RBD in a soluble form (wild-type and delta variant) [33]. However, this fusion tag does not necessarily result in correct disulfide bond formation. To overcome this problem, we expressed the MBP-RBD protein in the *Shuffle-T7* (NEB) strain, and after an amylose-based affinity chromatography step, we applied an additional refolding step to get correctly folded protein suitable for BLI-based binding measurements [34,35].

### 3.2. Production and Purification of Ecotin–Nanobody Fusion Proteins

To produce the ecotin–nanobody fusion protein, different expression conditions (induction time, temperature, media type, etc.) were tried (Supplementary Information, Tables S1–S3).

Media and additives have an impact on protein expression, so first, different types of media were probed. LB, a conventional rich medium, or an M9 minimal medium resulted in low yield, and interestingly, the usage of autoinduction media produced no detectable expressed fusion protein. In contrast, if the 2YT medium, a nutrient-rich microbial broth, was used, the fused nanobodies had good yield in different bacterial strains.

Different inducer concentrations were also explored since protein activity and expression capacity are sensitive to this. Induction with a 0.5 mM/L final concentration of IPTG proved to give the highest yield.

Then, several incubation temperatures (18, 23, 30 and 37 °C) and incubation times (4–12 h) were tested after adding IPTG into the 2YT culture medium. The best yield was reached when the expression occurred for a longer time (12 h) at a moderately high temperature (30 °C).

Expression of ecotin fusion proteins was attempted using two different promoters. When the promoters were tested, the T7-promoter-based (pET) construct resulted in low yield even when different expression conditions (*T* and *t*) were applied, both in the periplasm of *C43(DE3)* cells and the cytoplasm of *Shuffle-T7* cells, compared to the Tac-promoter-bases construct.

The best yields (Table 1) were obtained by using Tac-promoter-based expression vectors with expression conditions as follows: each bacterial strain except *BL21(DE3)* (*C43(DE3), Shuffle-T7,* and *HB2151*) harboring the recombinant constructs were grown in 2YT media and protein production was induced at the exponential growing phase. After expression for 12 h at 30 °C, the cells were harvested by centrifugation and further purified (Section 2).

**Figure 2:**
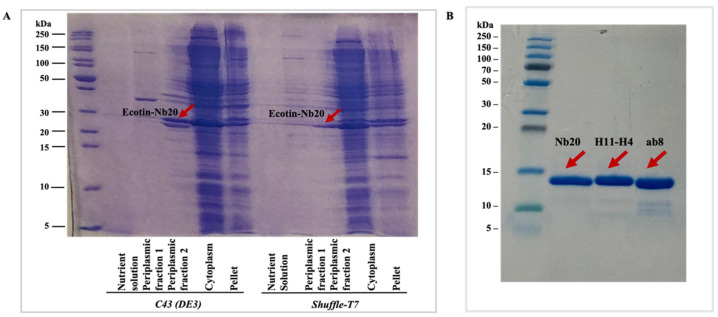
(**A**) Comparison of the fractions after periplasmic isolation (periplasmic isolation step one and step two), cytoplasm ultrasonication, and assessment of the nutrient solution by SDS-PAGE in the case of the expression of the Nb20 nanobody in *C43(DE3)* and *Shuffle-T7* bacterial strains using the Tac-promoter-based construct. Each sample contains 2.5 μL of sample mixed with 2x SDS loading dye. (**B**) Comparison of the purified nanobodies by SDS-PAGE in the case of the expression in *Shuffle-T7* bacterial strain using the Tac-promoter-based construct. Each sample contains 2.5 μL of sample mixed with 2x SDS loading dye.

**Figure 3:**
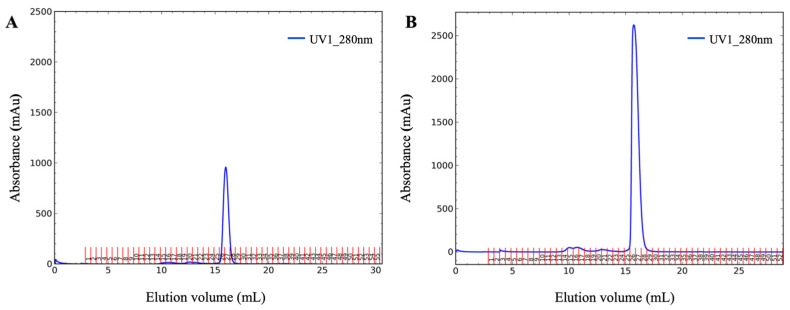
Size-exclusion chromatograms as the last purification step of the expression of the Nb20 nanobody from the (**A**) *C43(DE3)* bacterial strain periplasmic fraction and (**B**) *Shuffle-T7* bacterial strain from the cytoplasmic fraction. By comparing the size-exclusion chromatography results, the expression yield was much higher when the *Shuffle-T7* strain was used, which was expected. The amount of the starting bacterial pellet, after expression in one liter of nutrition solution, was the same in each case.

The fusion-protein-containing fractions from the cytoplasmic and periplasmic phase as well as the chromatography steps were detected by sodium dodecyl sulfate-polyacrylamide gel electrophoresis (SDS-PAGE) (Figure 2A). The yields were calculated as the summarized concentration (measured by absorbance at 280 nm) of the target nanobody-containing fractions after the SEC step (Figure 3), and LC-MS analysis was performed for each of the nanobodies (Figures S1–S3) to demonstrate the purity of the samples. Additionally, SDS-PAGE was run to detect the purified nanobodies (Figure 2B).

It can be easily seen that by using the Tac promoter, depending on the strain, the fusion protein could be purified from the periplasmic and/or cytoplasmic fraction (Table 1). The periplasmic targeting and isolation of the nanobodies was only successful in the case of the *C43(DE3)* bacterial strain. The fusion protein could be isolated both from the periplasmic and cytoplasmic fraction in the case of *HB2151* and *Shuffle-T7* cells. Though the ecotin-fused protein was not fully targeted into the periplasm, the expression yield was much higher in the *Shuffle-T7* cytoplasm than in the *C43(DE3)* periplasm, which is not surprising (Figure 3). However, if we compare the yields with the various preparations including the trial expressions (Supplementary Information), the highest yield could be achieved from the cytoplasm of the *Shuffle-T7* strain. It is important to note that the highest yield was achieved only when ecotin was present and was fused to the proteins of interest. This suggests that the ecotin fusion tag helps to keep the nanobodies in a soluble form and that it may slow down protein folding, which results a correct fold at the end.

If we compare the different bacterial strains, the *C43(DE3)* strain gave the best result for periplasmic expression, and using this strain, all the selected nanobodies were successfully isolated from this compartment. The *Shuffle-T7* strain gave good results as well, in spite of nanobodies being expressed in the cytoplasm, because their correct folding could be ensured by cytoplasmic DsbC, a disulfide bond isomerase. Expression in the *HB2151* strain gives variable results regarding the expression of different nanobodies, so this strain is not as suitable for the development of a generally applicable nanobody production protocol. The *BL21(DE3)* strain may also not be suitable, as the expressions surprisingly did not work in this strain (Supplementary Information).

### 3.3. Production and Purification of the SARS-CoV-2 Spike RBD Variants Fused to the MBP Fusion Protein

In case of the RBD domains, protein production was performed using 2YT medium. Induction of recombinant protein synthesis was initiated at the exponential phase. After expression, the cytoplasmic fraction was further purified by affinity chromatography, and before the final SEC purification step, the sample was incubated with 1 mM EDTA to avoid non-specific protease cleavage as well as with 10 mM DTT to reduce the extent of incorrectly formed disulfide bonds in the RBD domain. Then the protein was refolded by the slow-drop method, and for the final SEC step, a HiLoad Superdex75 column was used (Supplementary Information Figure S4). The expression yield in this case was 4.4 mg/L expression for the wild-type RBD-MBP and 4.1 mg/L expression for the delta variant RBD-MBP.

### 3.4. Validating the Structure and Functionality of the Recombinantly Expressed SARS-CoV-2 Spike RBD Domains

The correct 3D structure of refolded and non-refolded RBD samples were further examined by electronic circular dichroism (ECD) spectroscopy. The results show that at the aromatic region (255–275 nm), they have a similar fine structure, but minor differences indicate that the refolded and non-refolded samples have a somewhat different secondary fold (Supplementary Information Figures S5 and S6) [36].

### 3.5. Validating the Structure and Functionality of the Recombinantly Expressed Nanobodies

**Figure 4:**
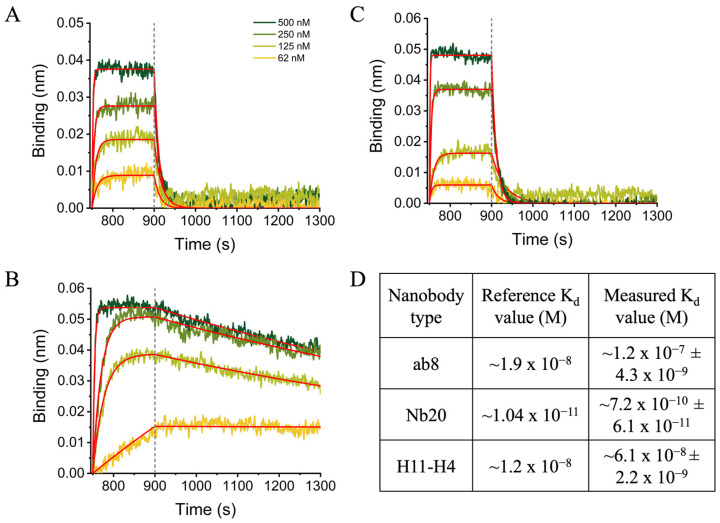
Binding of (**A**) ab8; (**B**) Nb20; and (**C**) H11-H4 anti-Spike antibody variants to the full-length Spike protein at different concentrations. The dissociation of the Nb20-Spike protein complex is much slower compared to ab8 and H11-H4, explaining the higher affinity of Nb20 to the Spike protein (K_d_~1 nM). Dashed lines represent the end of association. Table (**D**) contains the collected reference and measured K_d_ values.

To detect the functionality of the bacterially produced nanobodies, we studied their binding to the full-length spike protein and RBD of COVID-19 (S-protein) using BLI measurements (Figure 4 and Figure S7). All the purified nanobodies from *E. coli* cells were found to bind strongly to the RBD domain of the S protein with K_d_ values close to the previously published data, independently of the production and isolation method (Figure 4D) [9,18,19].

The observed slight differences compared to their human IgG counterparts might be related to the fact that full-length IgG molecules contain two identical binding sites on one molecule, while nanobodies contain only one. Moreover, they are glycosylated, while the nanobodies are not. However, the relative differences between the different nanobodies nicely coincide with the reference K_d_ values showing that Nb20 is the strongest binder among the nanobodies studied. The lower K_d_ value observed for Nb20 is due to its very slow dissociation compared to the others. It was previously reported that ab8 and H11-H4 nanobodies bind and release the S protein faster, indicating a mostly ionic interaction between the two partners [19,20]. On the other hand, the slow dissociation of Nb20 indicates hydrophobic interaction and more stable complex [18]. Nevertheless, our results clearly show that the nanobodies expressed in bacterial systems under different conditions are folded sufficiently to be functional. All the purified nanobodies from *E. coli* cells were found to bind to the RBD domain of the S protein with K_d_ values (10 nM) close to the previously published data, independently of the production and isolation method (Figure 4).

## 4. Conclusions

Nanobodies are attractive for the development of different diagnostic and therapeutic applications because of their beneficial biochemical properties such as their high antigen-binding affinity, high solubility, and stability. Here we describe a novel, ecotin-based fusion expression system, which allows their production in an easy and cost-effective way within four days. This system allows, with a rightly chosen strain and some optimization, the production of any target nanobody in the periplasm or in the cytoplasm, resulting in high-yield production of these nanobodies in a bioactive and well-folded form.

For different nanobody development platforms, this ecotin-fused nanobody expression system is an easily adoptable wet-lab method for the biophysical interaction analysis (BLI, SPR) of the engineered nanobodies.

## Figures and Tables

**Table 1:** Comparison of the expression yields (mg/L) by using different promoters (T7 and Tac) for the expressed Nb20 nanobody in *C43(DE3)* and *Shuffle-T7* bacterial strains and comparison of the yields of the different nanobodies. Protein synthesis occurred predominantly either in the cytoplasm or periplasm by using different bacterial strains (*Shuffle-T7, C43(DE3),* and *HB2151*) and the Tac promoter. * cp: cytoplasm; pp: periplasmic space.

Nanobody Cell Line/Expression Compartment */Promoter	Nb20	ab8	H11-H4
*C43(DE3)*/pp/T7	0.176	-	-
*C43(DE3)*/pp/Tac	0.5	0.485	0
*Shuffle-T7*/cp/T7	0.363	-	-
*Shuffle-T7*/cp/Tac	3.687	2.394	0.831
*HB2151*/cp/Tac	3.784	7.686	0.862

## Data Availability

Not applicable.

## Supplementary Materials

The following supporting information can be downloaded at: https://www.mdpi.com/article/10.3390/bioengineering10030389/s1, Figure S1: HPLC chromatogram and MS spectrum of the ab8 nanobody; Figure S2: HPLC chromatogram and MS spectrum of the H11-H4 nanobody; Figure S3: HPLC chromatogram and MS spectrum of the Nb20 nanobody; Figure S4: Comparison of chromatograms from the size-exclusion chromatography of refolded and non-refolded spike RBD delta variants; Figure S5: Comparison of spike RBD delta and wild-type variants after a refolded and non-refolded purification process by NUV-ECD measurements; Figure S6: Comparison of spike RBD delta and wild-type variants after a refolded and non-refolded purification process by FUV-ECD measurements; Figure S7: Binding of the VHH72 and Nb20 anti-spike antibody variants to the receptor-binding domain of the spike protein fused to the maltose-binding protein at different concentrations; Table S1: The VHH72 nanobody expression trials under different conditions; Table S2: The Nb20 nanobody expression trials under different conditions; Table S3: The ab8 nanobody expression trials under different conditions.

## Abbreviations

ACE2: angiotensin-converting enzyme 2, BLI: biolayer interferometry, CV: column volume, *E. coli*: *Escherichia coli*, FUV-ECD: far-UV electronic circular dichroism, Fv: variable fragment, IgG: immunoglobulin, IPTG: isopropyl β-D-1-thiogalactopyranoside, K_d_: dissociation constant, lac1: operon type, LPP: lipoprotein, MBP: maltose-binding protein, Ni-IMAC: Ni-immobilized metal affinity chromatography, NusA: N-utilization substance, NUV-ECD: near-UV electronic circular dichroism, rAb: recombinant antibody, RBD: receptor-binding domain of S protein, SARS-CoV-2: severe acute respiratory syndrome coronavirus 2, scFab: single chain antibody fragment, scFv: single chain variable fragment, sdAb: single domain antibody, TrxA: thioredoxin, V_H_-Fc ab8: neutralizing nanobody type, V_H_: variable heavy, VHH: heavy chain antibodies, V_L_: variable light.
