# Supplementary material for: A Novel Fusion Protein System for the Production of Nanobodies and the SARS-CoV-2 Spike RBD in a Bacterial System
Source: Bioengineering (Basel). 2023 Mar 22;10(3):389. doi: 10.3390/bioengineering10030389 (PMC10045489; doi:10.3390/bioengineering10030389)
Supplement: Supplementary file 1 [file bioengineering-10-00389-s001.zip › bioengineering-2280155-supplementary.pdf]

Supplementary Information

DNA sequence with the ecotin fusion protein sequence (NdeI and XhoI restriction sites are highlighted):

cat atg aag acg atc tta cct gct gtg ctt ttc gct gct ttt gca acg act agt gcc tgg gcc gca gaa tct gtt cag cct ctg gaa aaa atc gcg cct tat ccc cag gct gaa aag ggc atg aaa cgc caa gtc atc caa ctt acc ccg caa gag gat gaa tcg acc ttg aaa gtg gaa ctg tta atc ggg cag aca tta gag gtt gac tgc aac ctt cat cgt ctt ggc ggc aaa tta gag aac aag acc ttg gag gga tgg ggg tac gac tac tat gtg ttt gat aag gtc agt tct cca gtc tcg act atg atg gct tgc cct gat ggg aaa aag gag aag aag ttt gtt acc gcc tat tta ggc gac gct gga atg ctt cgc tac aac agt aag tta ccc atc gtg gtt tat acg ccc gat aat gtt gac gtt aaa tac cgc gtt tgg aag gca gaa gag aaa atc gac aat gcg gtt gtg cgc gga tca act agt ggt tct ggt cat cac cat cac cat cac tcc gcg ggt ctg gtg cca cgc gga tcc gaa ttc gag ctc cgt cga caa gct tgc ggc cgc **ctc gag**

DNA sequence of the expressed nanobodies:

Nb20:

ggt tct cag gtt cag ctg gtt gaa tct ggt ggt ggt ctg gtt cag gcg ggt ggt tct ctg cgt ctg tct tgc gcg gtt tct ggt gcg ggt gcg cac cgt gtt ggt tgg ttc cgt cgt gcg ccg ggt aaa gaa cgt gaa ttc gtt gcg gcg atc ggt gcg tct ggt ggt atg acc aac tac ctg gac tct gtt aaa ggt cgt ttc acc atc tct cgt gac aac gcg aaa aac acc atc tac ctg cag atg aac tct ctg aaa ccg cag gac acc gcg gtt tac tac tgc gcg gcg cgt gac atc gaa acc gcg gaa tac atc tac tgg ggt cag ggt acc cag gtt acc gtt tct tct

ab8:

gaa gtt cag ctg gtt gaa tct ggt ggt ggt ctg gtt cag ccg ggt ggt tct ctg cgt ctg tct tgc gcg gcg tct ggt ttc acc ttc gac gac tac gcg atg tct tgg gtt cgt cag gcg ccg ggt aaa ggt ctg gaa tgg atc ggt cgt atg tac aac aac ggt cgt acc tct tac aac ccg tct ctg aaa tct ctg gtt acc atc tct cgt gac aac tct aaa aac acc ctg tac ctg cag atg aac tct ctg cgt gcg gaa gac acc gcg acc tac tac tgc gcg cgt gac aac ctg ggt tac cgt ccg tct gaa aac ctg tac ggt atg gac gtt tgg ggt cag ggt acc acc gtt acc gtt tct tct

H11-H4:

cag gtt cag ctg gtt gaa tct ggt ggt ggt ctg atg cag gcg ggt ggt tct ctg cgt ctg tct tgc gcg gtt tct ggt cgt acc ttc tct acc gcg gcg atg ggt tgg ttc cgt cag gcg ccg ggt aaa gaa cgt gaa ttc gtt gcg gcg atc cgt tgg tct ggt ggt tct gcg tac tac gcg gac tct gtt aaa ggt cgt ttc acc atc tct cgt gac aaa gcg aaa aac acc gtt tac ctg cag atg aac tct ctg aaa tac gaa gac acc gcg gtt tac tac tgc gcg cag acc cac tac gtt tct tac ctg ctg tct gac tac gcg acc tgg ccg tac gac tac tgg ggt cag ggt acc cag gtt acc gtt tct tct

VHH72:

caa gtg caa tta caa gaa agt gga ggt gga ctg gtg caa gct ggt gga tca tta cgt ctg tcc tgc gcg gct tca ggc cgc act ttt agt gaa tat gcg atg ggt tgg ttc cgc cag gcc cca ggc aaa gaa cgt gaa ttt gta gca act att tcg tgg agt ggg ggt agc aca tac tat aca gat agc gta aaa gga cgc ttc act att tct cgc gat aac gcc aag aac act gta tat ctg cag atg aat agc ctg aag cca gat gac aca gca gtt tat tac tgt gct gcc gcg ggc ctg gga acg gtg gtc agc gaa tgg gat tac gat tat gat tac tgg ggt cag ggt act cag gtg act gtg agt agt ggc tcc

DNA sequence of the full spike protein:

atg ttc gtg ttt ctg gtg ctg ctg cct ctg gtg tcc agc cag tgt gtg aac ctg acc aca aga acc cag ctg cct cca gcc tac acc aac agc ttt acc aga ggc gtg tac tac ccc gac aag gtg ttc aga tcc agc gtg ctg cac tct acc cag gac ctg ttc ctg cct ttc ttc agc aac gtg acc tgg ttc cac gcc atc cac gtg tcc ggc acc aat ggc acc aag aga ttc gac aac ccc gtg ctg ccc ttc aac gac ggg gtg tac ttt gcc agc acc gag aag tcc aac atc atc aga ggc tgg atc ttc ggc acc aca ctg gac agc aag acc cag agc ctg ctg atc gtg aac aac gcc acc aac gtg gtc atc aaa gtg tgc gag ttc cag ttc tgc aac gac ccc ttc ctg ggc gtc tac tat cac aag aac aac aag agc tgg atg gaa agc gag ttc cgg gtg tac agc agc gcc aac aac tgc acc ttc gag tac gtg tcc cag cct ttc ctg atg gac ctg gaa ggc aag cag ggc aac ttc aag aac ctg cgc gag ttc gtg ttc aag aac atc gac ggc tac ttc aag atc tac agc aag cac acc cct atc aac ctc gtg cgg gat ctg cct cag ggc ttc tct gct ctg gaa ccc ctg gtg gat ctg ccc atc ggc atc aac atc acc cgg ttt cag aca ctg ctg gcc ctg cac aga agc tac ctg aca cct ggc gat agc agc agc gga tgg aca gct ggt gcc gcc gct tac

tat gtg ggc tac ctg cag cct aga acc ttt ctg ctg aag tac aac gag aac ggc acc atc acc gac gcc gtg gat tgt gct ctg gat cct ctg agc gag aca aag tgc acc ctg aag tcc ttc acc gtg gaa aag ggc atc tac cag acc agc aac ttc cgg gtg cag ccc acc gaa tcc atc gtg cgg ttc ccc aat atc acc aat ctg tgc ccc ttc ggc gag gtg ttc aat gcc acc aga ttc gcc tct gtg tac gcc tgg aac cgg aag cgg atc agc aat tgc gtg gcc gac tac tcc gtg ctg tac aac tcc gcc agc ttc agc acc ttc aag tgc tac ggc gtg tcc cct acc aag ctg aac gac ctg tgc ttc aca aac gtg tac gcc gac agc ttc gtg atc cgg gga gat gaa gtg cgg cag att gcc cct gga cag aca ggc aag atc gcc gac tac aac tac aag ctg ccc gac gac ttc acc ggc tgt gtg att gcc tgg aac agc aac aac ctg gac tcc aaa gtc ggc ggc aac tac aat tac ctg tac cgg ctg ttc cgg aag tcc aat ctg aag ccc ttc gag cgg gac atc tcc acc gag atc tat cag gcc ggc agc acc cct tgt aac ggc gtg gaa ggc ttc aac tgc tac ttc cca ctg cag tcc tac ggc ttt cag ccc aca aat ggc gtg ggc tat cag ccc tac aga gtg gtg gtg ctg agc ttc gaa ctg ctg cat gcc cct gcc aca gtg tgc ggc cct aag aaa agc acc aat ctc gtg aag aac aaa tgc gtg aac ttc aac ttc aac ggc ctg acc ggc acc ggc gtg ctg aca gag agc aac aag aag ttc ctg cca ttc cag cag ttt ggc cgg gat atc gcc gat acc aca gac gcc gtt aga gat ccc cag aca ctg gaa atc ctg gac atc acc cct tgc agc ttc ggc gga gtg tct gtg atc acc cct ggc acc aac acc agc aat cag gtg gca gtg ctg tac cag gac gtg aac tgt acc gaa gtg ccc gtg gcc att cac gcc gat cag ctg aca cct aca tgg cgg gtg tac tcc acc ggc agc aat gtg ttt cag acc aga gcc ggc tgt ctg atc gga gcc gag cac gtg aac aat agc tac gag tgc gac atc ccc atc ggc gct ggc atc tgt gcc agc tac cag aca cag aca aac agc ccc gcc tct gtg gcc agc cag agc atc att gcc tac aca atg tct ctg ggc gcc gag aac agc gtg gcc tac tcc aac aac tct atc gct atc ccc acc aac ttc acc atc agc gtg acc aca gag atc ctg cct gtg tcc atg acc aag acc agc gtg gac tgc acc atg tac atc tgc ggc gat tcc acc gag tgc tcc aac ctg ctg ctg cag tac ggc agc ttc tgc acc cag ctg aat aga gcc ctg aca ggg atc gcc gtg gaa cag gac aag aac acc caa gag gtg ttc gcc caa gtg aag cag atc tac aag acc cct cct atc aag gac ttc ggc ggc ttc aat ttc agc cag att ctg ccc gat cct agc aag ccc agc aag cgg agc ttc atc gag gac ctg ctg ttc aac aaa gtg aca ctg gcc gac gcc ggc ttc atc aag cag tat ggc gat tgt ctg ggc gac att gcc gcc agg gat ctg att tgc gcc cag aag ttt aac gga ctg aca gtg ctg cct cct ctg ctg acc gat gag atg atc gcc cag tac aca tct gcc ctg ctg gcc ggc aca atc aca agc ggc tgg aca ttt gga gct ggc gcc gct ctg cag atc ccc ttt gct atg cag atg gcc tac cgg ttc aac ggc atc gga gtg acc cag aat gtg ctg tac gag aac cag aag ctg atc gcc aac cag ttc aac agc gcc atc ggc aag atc cag gac agc ctg agc agc aca gca agc gcc ctg gga aag ctg cag gac gtg gtc aac cag aat gcc cag gca ctg aac acc ctg gtc aag cag ctg tcc tcc aac ttc ggc gcc atc agc tct gtg ctg aac gat atc ctg agc aga ctg gac cct cct gaa gcc gag gtg cag atc gac aga ctg atc acc gga agg ctg cag tcc ctg cag acc tac gtt acc cag cag ctg atc aga gcc gcc gag att aga gcc tct gcc aat ctg gcc gcc acc aag atg tct gag tgt gtg ctg ggc cag agc aag aga gtg gac ttt tgc ggc aag ggc tac cac ctg atg agc ttc cct cag tct gcc cct cac ggc gtg gtg ttt ctg cac gtg aca tac gtg ccc gct caa gag aag aat ttc acc acc gct cca gcc atc tgc cac gac ggc aaa gcc cac ttt cct aga gaa ggc gtg ttc gtg tcc aac ggc acc cat tgg ttc gtg acc cag cgg aac ttc tac gag ccc cag atc atc acc acc gac aac acc ttc gtg tct ggc aac tgc gac gtc gtg atc ggc att gtg aac aat acc gtg tac gac cct ctg cag ccc gag ctg gac agc ttc aaa gag gaa ctg gat aag tac ttt aag aac cac aca agc ccc gac gtg gac ctg ggc gat atc agc gga atc aat gcc agc gtc gtg aac atc cag aaa gag atc gac cgg ctg aac gag gtg gcc aag aat ctg aac gag agc ctg atc gac ctg caa gaa ctg ggg aag tac gag cag tac atc aag tgg ccc

Amino acid sequence of the ecotin fusion protein:

MKTILPAVLFAAFATTSAWAAESVQPLEKIAPYPQAEKGMKRQVIQLTPQEDESTLKVELLIGQTLEVD
CNLHRLGGKLENKTLEGWGYDYYVFDKVSSPVSTMMACPDGKKEKKFVTAYLGDAGMLRYNSKLPI
VVYTPDNVDVKYRVWKAEEKIDNAVVRGSTSGSGHHHHHHSAGLVPR|GS

Amino acid sequence of the expressed nanobodies (residual amino acids from the cleaving are underlined):

Nb20:
GSQVQLVESGGGLVQAGGSLRLSCAVSGAGAHRVGWFRRAPGKEREFVAAIGASGGMTNYLDSVKG
RFTISRDNAKNTIYLQMNSLKPQDTAVYYCAARDIETAEYIYWGQGTQVTVSS

ab8:
GSQVQLVESGGGLVQPGGSLRLSCAASGFTFDDYAMSWVRQAPGKGLEWIGRMYNNGRTSYNPSLKS
LVTISRDNSKNTLYLQMNSLRAEDTATYYCARDNLGYRPSENLYGMDVWGQGTQVTVSS

H11-H4:
GSQVQLVESGGGLMQAGGSLRLSCAVSGRTFSTAAMGWFRQAPGKEREFVAAIRWSGGSAYYADSV
KGRFTISRDKAKNTVYLQMNSLKYEDTAVYYCAQTHYVSYLLSDYATWPYDYWGQGTQVTVSS

VHH72:
MDQVQLQESGGGLVQAGGSLRLSCAASGRTFSEYAMGWFRQAPGKEREFVATISWSGGSTYYTDSVK
GRFTISRDNAKNTVYLQMNSLKPDDTAVYYCAAAGLGTVVSEWDYDYDYWGQGTQVTVSS

Nanobody expression trials under different conditions:

Table S1. VHH72

Number of trials	Nutrition solution	Bacterial strain	Signal seq.	Prom.	Fusion protein	Expression compartment	Temp. (°C)	Yield (mg/L)
1.	Auto induction	*BL21 (DE3)*	-	T7	-	periplasm	18	0.17
2.	2YT	*BL21 (DE3)*	-	T7	-	periplasm	18	0
3.	2YT	*Shuffle-T7*	-	T7	-	periplasm	18	0
4.	2YT	*Shuffle-T7*	pelB	T7	-	periplasm	18	0.1
5.	2YT	*BL21 (DE3)*	-	T7	MBP	cytoplasm	18	0
6.	2YT	*Shuffle-T7*	-	T7	MBP	cytoplasm	18	0
7.	2YT	*BL21 (DE3)*	-	Tac	GST	cytoplasm	18	0
8.	2YT	*Shuffle-T7*	-	Tac	GST	cytoplasm	18	0
9.	Auto induction	*BL21 (DE3)*	-	T7	-	cytoplasm, periplasm	23	0
10.	Auto induction	*BL21 (DE3)*	-	T7	-	cytoplasm, periplasm	30	0
11.	Auto induction	*HB2151*	-	T7	-	cytoplasm, periplasm	30	0

Table S2. Nb20

Number of trials	Nutrition solution	Bacterial strain	Signal seq.	Prom.	Fusion protein	Expression compartment	Temp. (°C)	Yield (mg/L)
1.	2YT	*Shuffle-T7*	-	T7	-	cytoplasm	18	0
2.	2YT	*BL21 (DE3)*	-	T7	-	cytoplasm	18	0

Table S3. ab8

Number of trials	Nutrition solution	Bacterial strain	Signal seq.	Prom.	Fusion protein	Expression compartment	Temp. (°C)	Yield (mg/L)
1.	Auto induction	*HB2151*	-	T7	-	cytoplasm, periplasm	30	0
2.	2YT	*HB2151*	-	T7	-	cytoplasm, periplasm	30	0

Supplemental Figures:

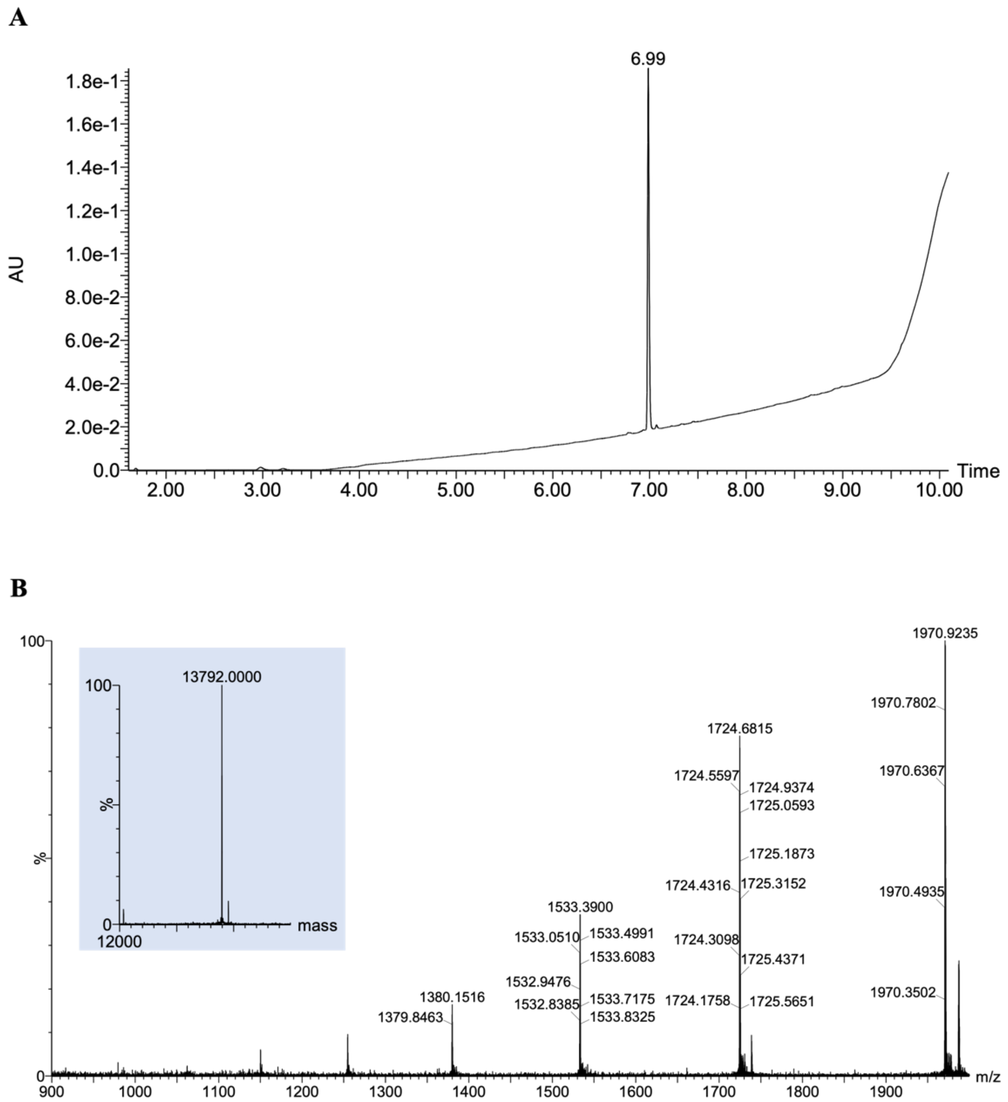

Figure S1. A) HPLC chromatogram (A_{220nm}) of the expressed ab8 nanobody. **B)** Mass spectrum and deconvoluted spectrum of the peak Rt=6.99 min.

A

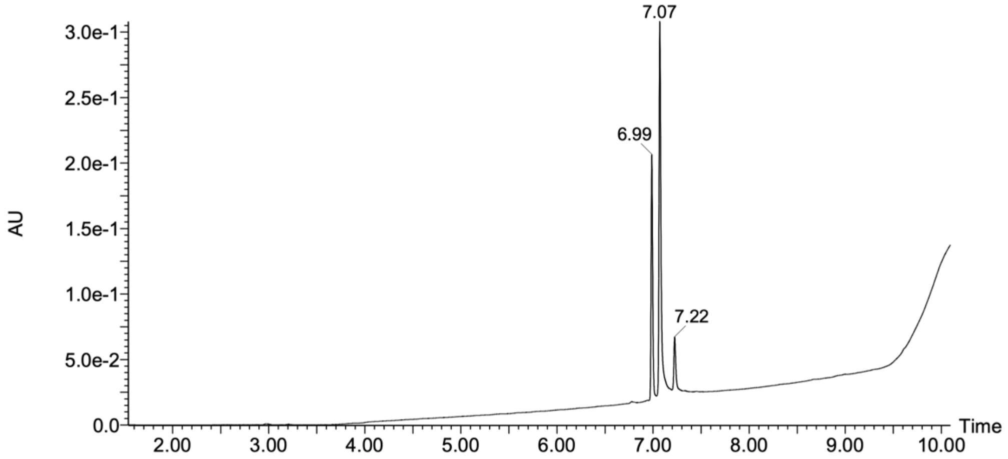
7.07
6.99
7.22
AU
Time
3.0e-1
2.5e-1
2.0e-1
1.5e-1
1.0e-1
5.0e-2
0.0
2.00
3.00
4.00
5.00
6.00
7.00
8.00
9.00
10.00

B

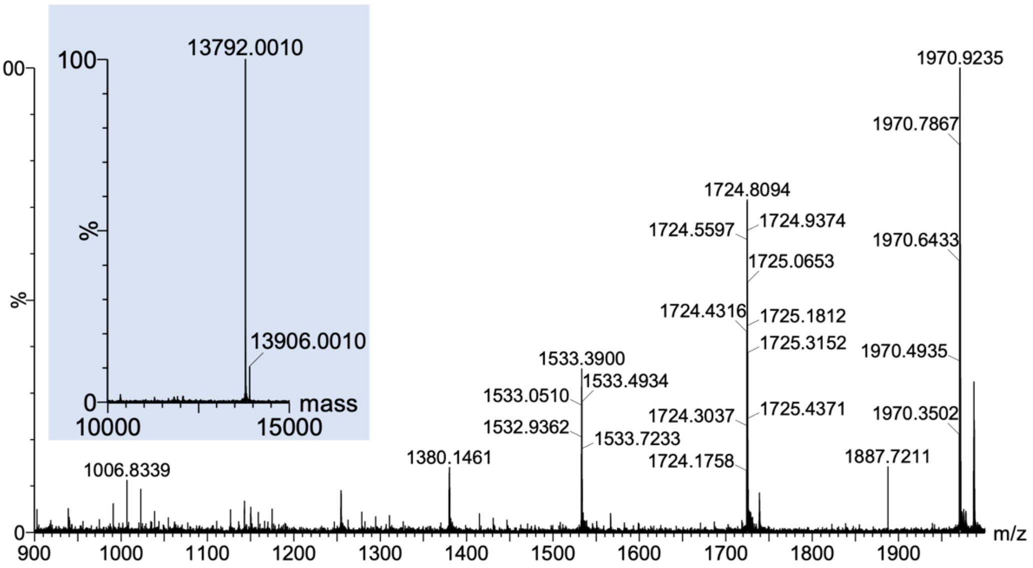
13792.0010
13906.0010
%
mass
10000
15000
1970.9235
1970.7867
1724.8094
1724.5597
1724.9374
1970.6433
1725.0653
1724.4316
1725.1812
1725.3152
1970.4935
1533.3900
1533.4934
1533.0510
1724.3037
1725.4371
1970.3502
1532.9362
1533.7233
1724.1758
1887.7211
1380.1461
1006.8339
%
m/z

C

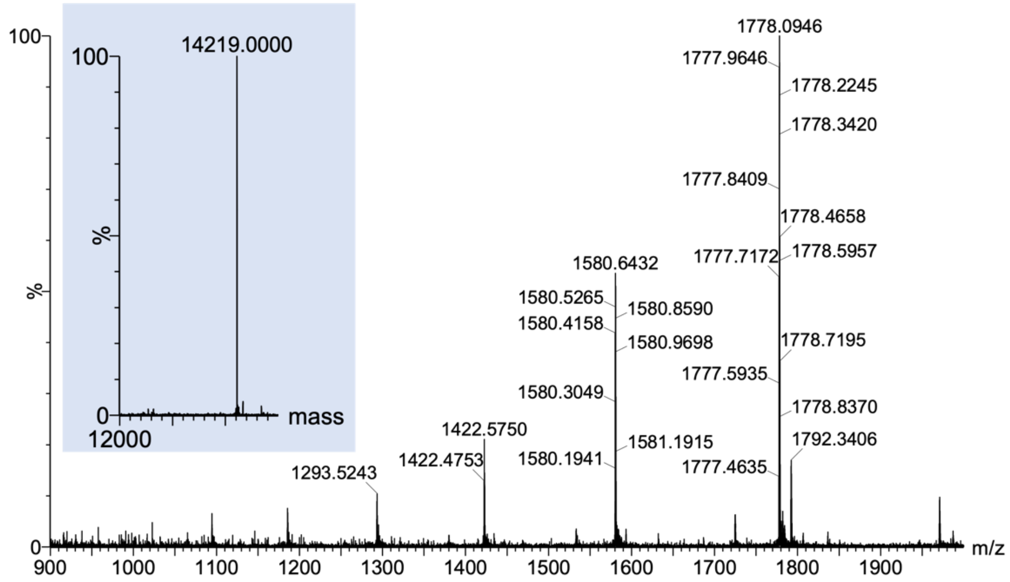

D

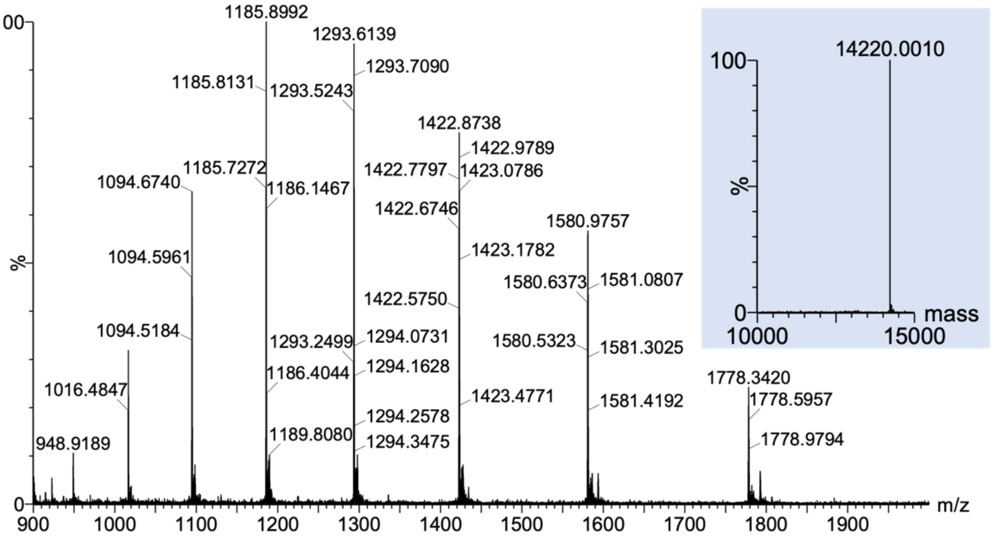

Figure S2. A) HPLC chromatogram (A_{220nm}) of the expressed H11-H4 nanobody. **B)** Mass spectrum and deconvoluted spectrum of the peak Rt=6.99 min. **C)** Mass spectrum and deconvoluted spectrum of the peak Rt=7.07 min. **D)** Mass spectrum and deconvoluted spectrum of the peak Rt=7.22 min.

A

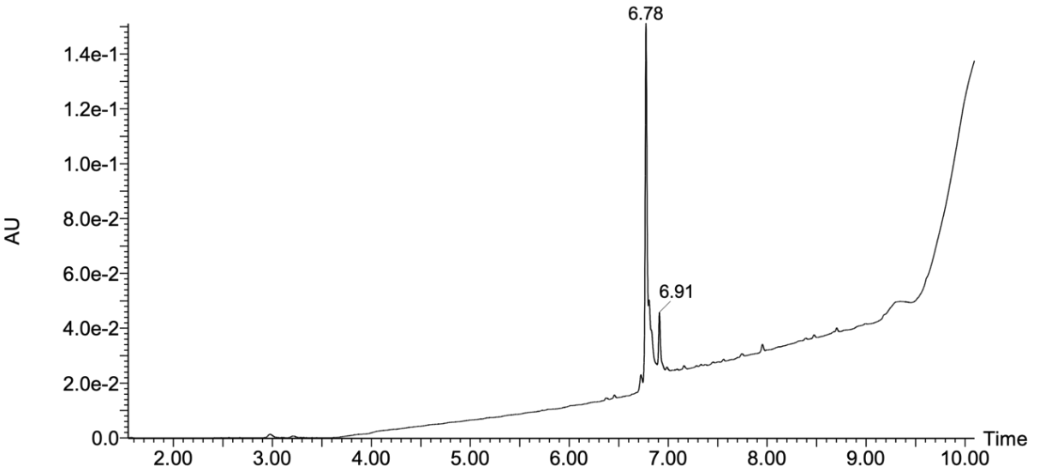

6.78
6.91
AU
1.4e-1
1.2e-1
1.0e-1
8.0e-2
6.0e-2
4.0e-2
2.0e-2
0.0
2.00
3.00
4.00
5.00
6.00
7.00
8.00
9.00
10.00
Time

B

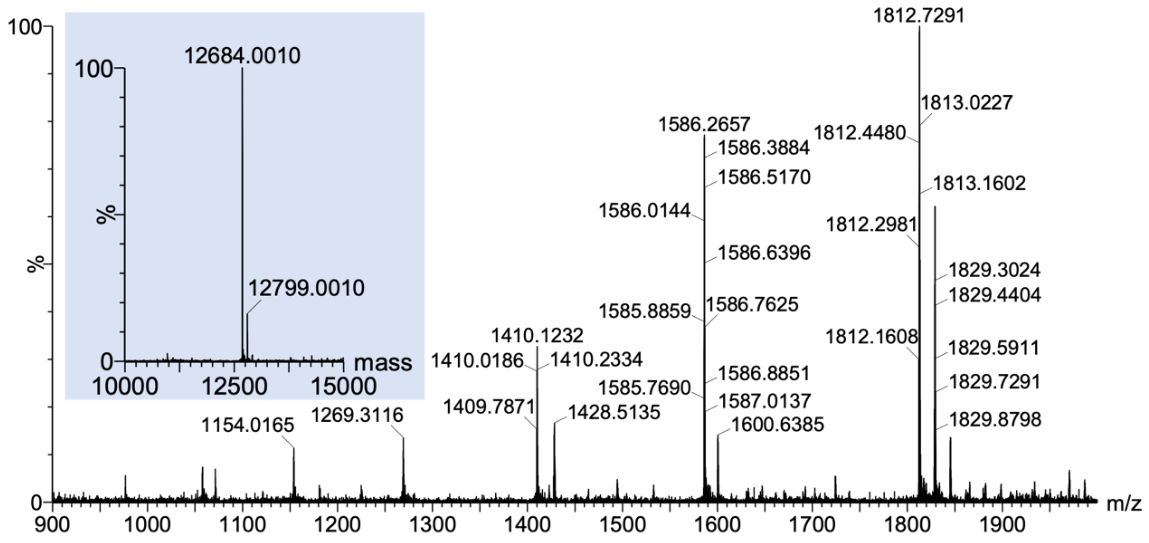

12684.0010
12799.0010
100
%
0
10000
12500
15000
mass
1812.7291
1813.0227
1812.4480
1813.1602
1812.2981
1812.1608
1829.3024
1829.4404
1829.5911
1829.7291
1829.8798
1586.2657
1586.3884
1586.5170
1586.0144
1586.6396
1585.8859
1586.7625
1586.8851
1585.7690
1587.0137
1600.6385
1410.1232
1410.0186
1410.2334
1409.7871
1428.5135
1154.0165
1269.3116
m/z
900
1000
1100
1200
1300
1400
1500
1600
1700
1800
1900

C

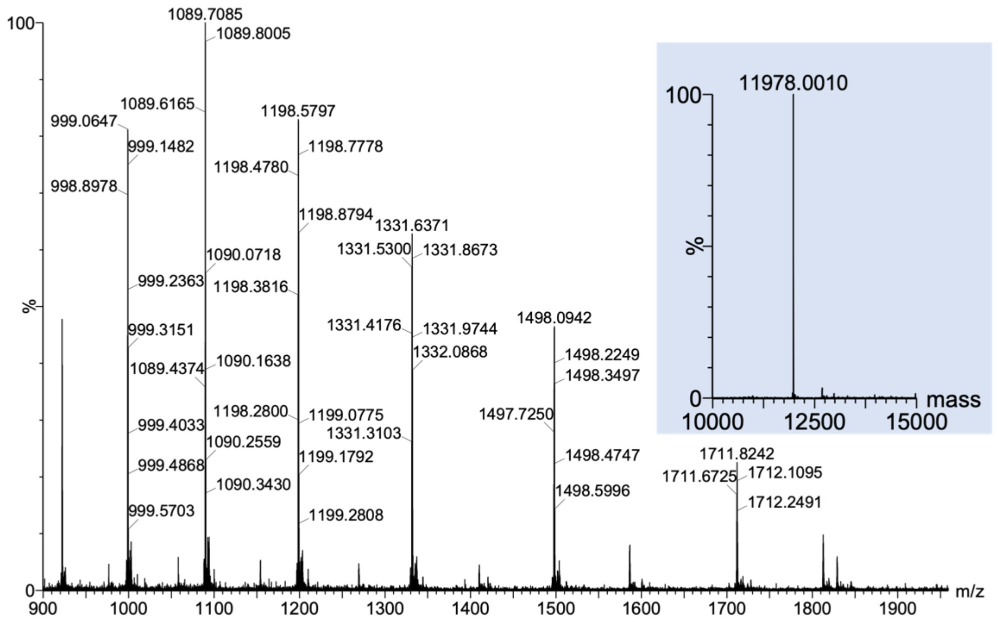

Figure S3. A) HPLC chromatogram (A_{220nm}) of the expressed Nb20 nanobody. **B)** Mass spectrum and deconvoluted spectrum of the peak Rt=6.78 min. **C)** Mass spectrum and deconvoluted spectrum of the peak Rt=6.91 min.

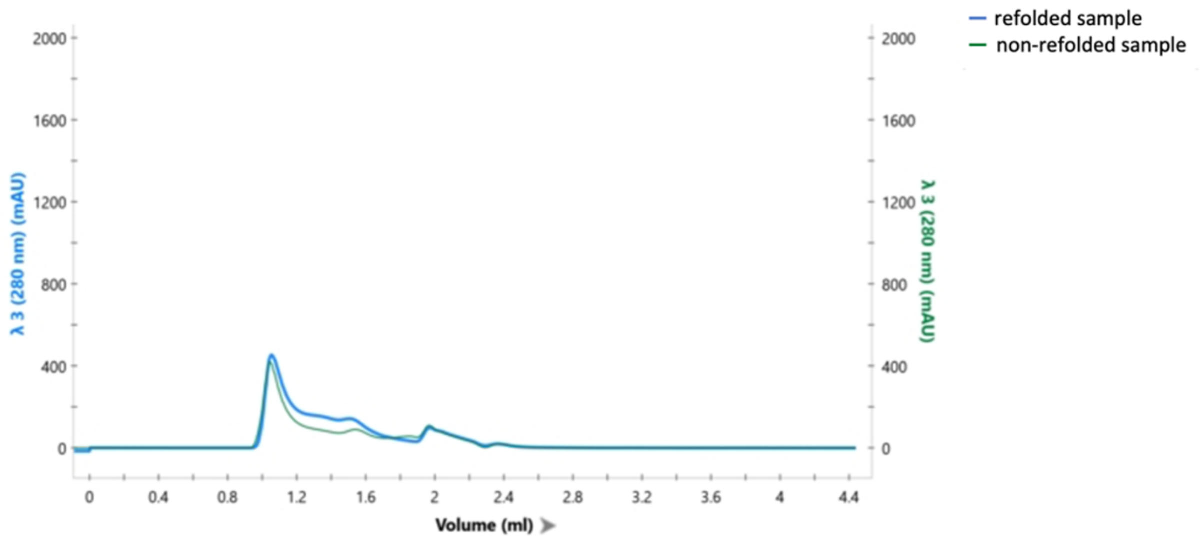

Figure S4. Comparison of chromatograms from the size-exclusion chromatography of refolded (blue) and non-refolded (green) spike RBD delta variants.

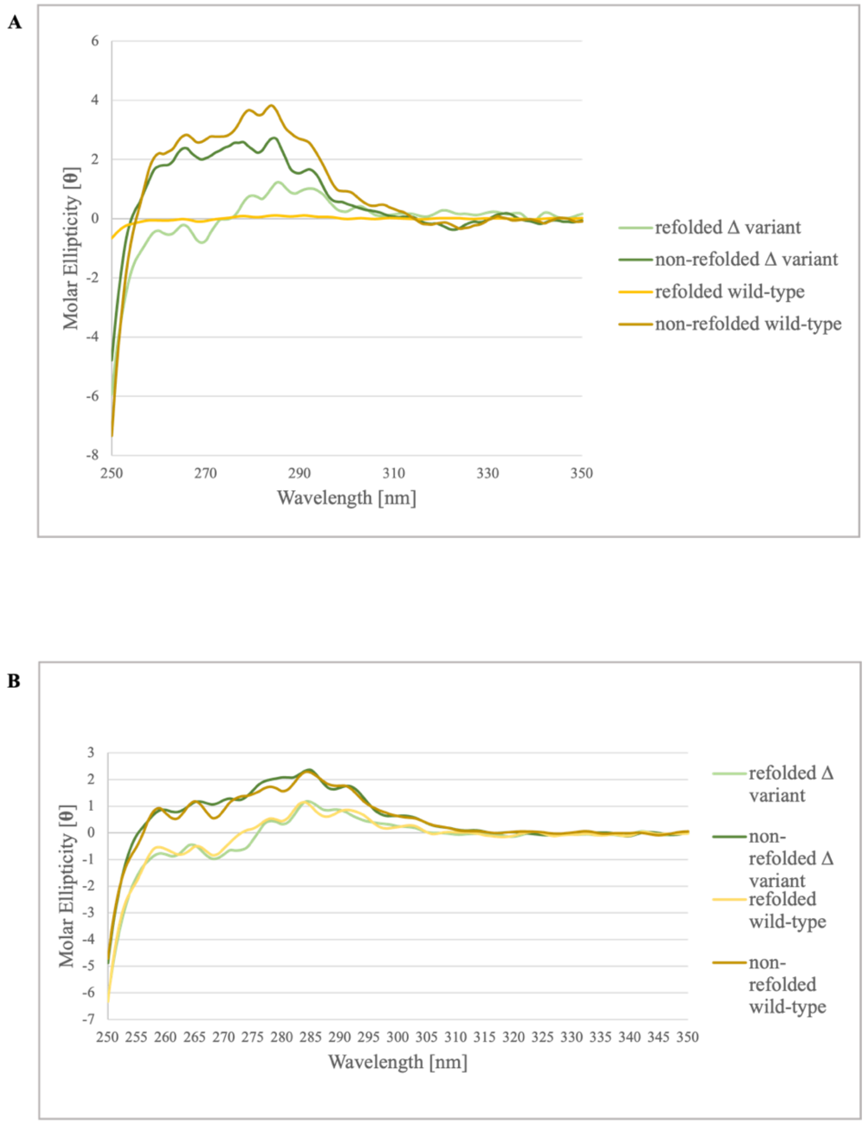

Figure S5. Comparison of spike RBD delta and wild-type variants after a refolded and non-refolded purification process by NUV-ECD measurements at **A)** 8µM and **B)** 16 µM concentrations.

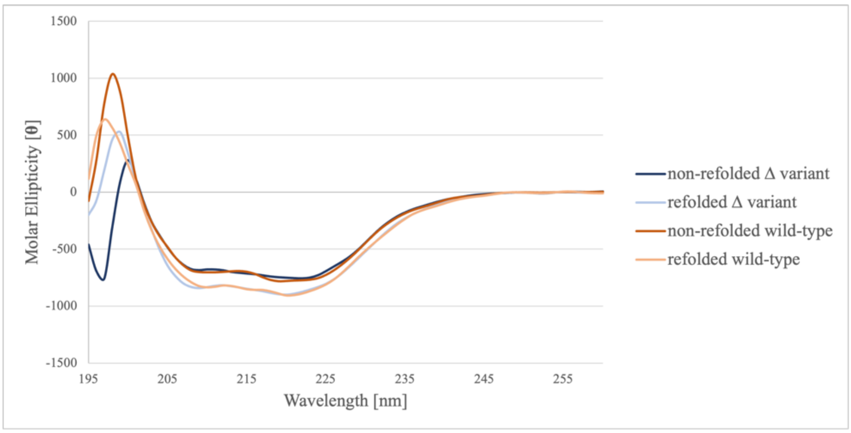

Figure S6. Comparison of spike RBD delta and wild-type variants after a refolded and non-refolded purification process by FUV-ECD measurements at a concentration of 16 µM.

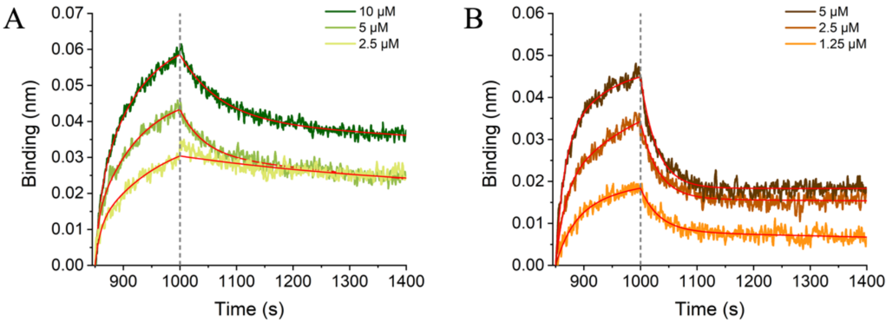

C

Nanobody type	Reference K_d value (M)	Measured K_d value (M)
Nb20	~1.04×10^{-11}	~$3.6 \times 10^{-8} \pm 8.7 \times 10^{-9}$
VHH72	~3.9×10^{-8}	~$3.1 \times 10^{-7} \pm 7.0 \times 10^{-9}$

Figure S7. Binding of **A)** VHH72 and **B)** Nb20 anti-spike antibody variants to the receptor-binding domain of the spike protein fused to the maltose-binding protein at different concentrations. Table **C** contains the collected reference [18,20] and measured K_d values**.**
